# Grab Bar Grasp Location During Bathtub Exit and Sit-to-Stand Transfers: Biomechanical Evaluation

**DOI:** 10.2196/69442

**Published:** 2025-07-22

**Authors:** Iris C Levine, Konika Nirmalanathan, Roger E Montgomery, Alison C Novak

**Affiliations:** 1 KITE Research Institute, University Health Network, 550 University Avenue, Toronto, ON, M5G 2A2, Canada, 1 4165973422 ext 7713; 2 Rehabilitation Sciences Institute, University of Toronto, Toronto, ON, Canada; 3 Faculty of Kinesiology and Physical Education, University of Toronto, Toronto, ON, Canada; 4 Department of Occupational Sciences and Occupational Therapy, University of Toronto, Toronto, ON, Canada

**Keywords:** bathroom, accessibility, accidental falls, home modification, bathtub exit

## Abstract

**Background:**

Grab bars are a multi-function bathing tool. While grab bars are commonly recommended by rehabilitation professionals, existing literature regarding optimal grab bar locations is focused on preference rather than function.

**Objective:**

This study aimed to evaluate grab bar grasp location on 8 grab bar configurations during bathtub exit, with and without balance loss, and sit-to-stand (STS) from a bath seat.

**Methods:**

Motion capture was used to evaluate grasp location during bathing activities in 28 older (65+ years) and 37 younger (18‐35 years) adults. Grasp location was compared between age groups and balance loss conditions using ANOVA, and correlated with body height.

**Results:**

Vertical grasp location varied from close to the bathtub rim to more than 1 meter above the bathtub rim (maximum 22.4 cm), while horizontal grasp location was close to the bathtub rim during bathtub exit, and close to the bath seat during STS. Young adult participants grasped 9.4% lower on vertical grab bars during perturbation trials than nonperturbation trials (*P*<.01). Body height was positively correlated with grasp height on a vertical grab bar during nonperturbation trials (*r*=0.67, *P*<.01), and negatively correlated with grasp distance on a low horizontal grab bar during STS (*r*=−0.37, *P*=.03).

**Conclusions:**

Grab bar grasp location varied between proactive and reactive grasp scenarios and was linked to user height for some situations. These findings may be used to guide the selection of a grab bar installation location to support multiple bathing tasks.

## Introduction

Grab bars serve multiple functions during bathing. While grab bars are a critical device for fall prevention [1-3], they are also used to support balance during bathtub entry and exit [4,5], and for tasks such as getting up from and sitting down on a bath seat [6].

Directing the installation of grab bars in bathing environments is a common task for occupational therapists and other rehabilitation professionals. However, there is a lack of agreement regarding the placement of the grab bars. Building codes [7] and accessibility standards [8] that exist to guide placement vary in their recommendations and are not necessarily based on empirical evidence supporting safety and usability. While tools exist to assist with the selection of assistive devices for bathing [9,10], there are no current tools to help occupational therapists decide where grab bars should be placed. Existing research on grab bar placement is primarily based on preference rather than effectiveness for balance support [3,11-13]. Sveistrup et al [11,14] found that users preferred configurations with grab bars positioned near the bathtub rim to assist with entry and exit transfers, but did not otherwise find differences between the configurations evaluated. Guitard et al [3] extended this work, finding that older adults prefer vertical grab bars for entry and exit. In contrast, Batista et al [12] found a preference for horizontal grab bars over vertical grab bars during a sit-to-stand (STS) task in younger adults. While these authors have presented findings regarding preferred grab bars, the researchers did not report where the participants grasped along the grab bar. It is unclear what grasp location is preferred to support bathing transfers and activities without balance loss, what grasp location is most effective for balance recovery, or whether the preferred grasp location differs from the reactive grasp location during balance loss.

Functionally, grab bars are installed for both accessibility and fall prevention purposes, but it is not clear if the same grab bar placement is appropriate for proactive use for mobility, and reactive use for fall prevention. During bathing tasks such as STS, grasp location has a demonstrated effect on ergonomic factors, such as task speed or muscle activation [15,16], as well as factors related to fall risk, such as greater trunk flexion, trunk angle, or forward center-of-mass (COM) position [15-18]. While stored visuospatial information may influence grasp location during unexpected balance perturbation [19], other factors may also contribute. Grasp height affects COM control and physical demands associated with balance recovery [20,21]. In a loss-of-balance scenario during bathing tasks, managing rapid COM motion, compensatory reactions, and underfoot friction, the grasp location may differ from under nonbalance loss conditions. Determining an appropriate grab bar location to support daily use, as well as unexpected balance loss, would be valuable to improve the utility of grab bars and guide installation recommendations.

Further, age may play a role in the optimal location for grab bar placement. Guitard et al [3] found differences in grab bar use strategies and balance recovery reactions between older and younger adults. In some cases, the younger adult cohort avoided using a grab bar at all, while the older adult cohort was more proactive in using grab bars and other support devices. As summarized by Maki et al [22], reach-to-grasp reactions to balance perturbances are slower and smaller in older adults than younger adults. Reach-to-grasp reactions are also more variable in older adults and more reliant on visual targeting of the handhold than in younger adults [23]. Shoulder range of motion decreases with age, limiting the comfortable grasp location of an older adult compared with a younger adult [24]. These factors may contribute to differing grasp location strategies between younger and older adults.

Therefore, the purpose of this research was to evaluate the grasp location on a grab bar during bathtub exit and STS tasks. As a secondary goal, we aimed to determine whether grasp location differed between grab bar configurations, non-balance-loss and perturbation bathtub exits, and between older and younger adults. Finally, we wanted to know whether grasp location was related to the participant’s body height.

## Methods

### Overview

Community-dwelling adults were recruited and consented to participate in either a bathtub exit task (EXIT) or a STS/stand-to-sit task with a bath seat (STS). For either study, participants were required to be able to enter and exit a bathtub independently and were excluded if they reported any mobility, orthopedic, or neurological conditions; used mobility aids; or had a recent injury (eg wrist sprain) or condition (eg, concussion or ear infection) that would affect their balance or ability to complete the protocol. Participants were grouped into older adults (65+ years) or younger adults (18‐35 years). For both protocols, in order to limit the effect of fatigue, we limited the study duration, permitted breaks and snacks, and frequently questioned participants about their energy levels and interest in continuation.

### Ethical Considerations

Both protocols (UHN REB# 17‐6236 and 18‐5406) were approved by an institutional research ethics board at the University Health Network. Participants provided written informed consent. Participant privacy and confidentiality were maintained throughout the study, and all data were deidentified. Participants were compensated for their time ($10 CAD per hour) and travel costs ($20 CAD). At the time of data collection, the compensation was approximately equivalent to US $7.75 and US $15.50, respectively.

### Environment, Equipment, and Materials

Data were collected in the General Purpose Lab of the Challenging Environment Assessment Laboratory. The laboratory is a modifiable 5.55 × 5.15m space, which can be affixed to a 6-degree-of-freedom motion base (Rexroth, Lohr am Main, Germany) to evoke perturbations. A custom-built weldment was designed to permit the installation of vertically-, horizontally-, and diagonally-oriented grab bars (3.8 cm diameter, enamel-coated steel). A customized bathtub was modified so that all edges of the bathtub were removed and replaced with a foam block (41 cm in height, coinciding with the rim height of the bathtub in its initial state) to replicate the bathtub’s rim. A plywood-backed white plastic sheet was placed behind the grab bars to reduce the contrast between the apparatus and the grab bars. For the STS protocol, a height-adjustable bath seat was placed at one end of the bathtub, so that when the participant was seated, the remainder of the bathtub would be in front of them, and the grab bars at their right.

A 15-camera motion capture system (200 Hz; Motion Analysis Corp, Santa Rosa, USA) was used to track wrist motion during EXIT and STS via reflective markers. Markers were placed on the right and left radial and ulnar styloid processes. Additional markers were placed on each end of the grab bar, on the outside corners of the bath seat, on the corners of the floor surrounding the bathtub, and along the bathtub rim.

One liter of diluted sodium lauryl sulfate (SLS) was poured into the bottom of the bathtub to simulate a realistic bathing environment. For STS, 1 g SLS per liter of water was used in accordance with the British Standard Institution slip-resistant test standard (BS EN 13845) to create a moderately slippery surface while maintaining the safety of participants with bathing difficulty. For EXIT, 2 g SLS/liter was used, as it more consistently resulted in slips during pilot testing.

EXIT participants donned a harness, tethered via a fall arrest line to a custom robotic overhead gantry (BerkelaarMRT, Delft, Netherlands) that permitted free motion, except in the case that the participant traveled downward >10cm. STS participants did not use a harness due to the lower risk of the task and the inability to move through a vertical space without the gantry locking. In both cases, participants wore knee and elbow pads. Additionally, EXIT participants wore a full-mask hockey helmet.

Finally, for EXIT with a perturbation, a laser sensor was affixed in line with the outer edge of the bathtub wall, such that when a participant lifted their leg over the wall and past the sensor, the perturbation (perpendicular to the direction of travel; 2m/s^2^; peak velocity: 0.4 m/s; peak displacement: 0.08 m [3]) was triggered. No perturbations were delivered during STS.

### Experimental Conditions

Several grab bar orientations were evaluated to represent a breadth of configurations in existing codes and standards or commonly used due to market availability or ease of installation. For the EXIT task, three grab bar orientations were evaluated: (1) a standard horizontal (SH) grab bar, 91.4 cm above the ground [25]; (2) a high horizontal (HH) grab bar at 102 cm above the ground (established as preferred [12] and superior for balance recovery [20]); and (3) a vertical (VRT) grab bar directly above the bathtub rim [7]. The EXIT task grab bars were placed along the side wall of the bathtub, centered above the bathtub rim. For the STS task, 5 grab bar orientations were evaluated; the grab bars were placed along the back (ie, long) wall of the bathtub. In addition to the SH and VRT grab bars, we also evaluated (1) a low horizontal (LH) grab bar 58.4 cm above the ground [7,25]; (2) a 45° incline (I45) grab bar (simulating a 24”/61.0 cm grab bar between two 16”/40.6 cm-spaced studs); and (3) a 60° incline (I60) grab bar (available in prefabricated angled designs). Each grab bar had a minimum graspable length of 1.2 m.

### EXIT Protocol

For the EXIT protocol, the participant was instructed to enter the bathtub, turn around within the tub, take 3 steps in place, and exit the bathtub. Participants were asked to perform the bathtub transfer several times using a self-selected strategy. Participants were aware that a perturbation would be delivered during one of the transfers but were not alerted as to which trial the perturbation would be delivered. All older adults self-selected to use a hand-in-place approach, placing their hand on the grab bar prior to completing the transfer (older adult, hand-in-place group, OA-HIP). Ten younger adults self-selected to use a reach-to-grasp approach, initially exiting the bathtub without using the grab bar and reaching to grasp the grab bar once the lead foot had passed the laser sensor (younger adult, reach-to-grasp group, YA-RTG). Ten younger adults were asked to use a hand-in-place approach for comparison with the OA-HIP group (younger adult, hand-in-place group, YA-HIP). Only the perturbation trials were analyzed for OA-HIP and YA-HIP (one per grab bar), as participants were unaware of the timing of the perturbation and were not primed to alter their grasp location. Perturbation and nonperturbation trials (one each, for each grab bar) were analyzed for YA-RTG participants.

### STS Protocol

For the STS protocol, the seat pan was adjusted to a comfortable height. The participant was asked to sit on the bath seat and use the grab bar to help them rise from the seat and return to a seated position. One trial was analyzed for each grab bar condition. Older adults and younger adults (YA-STS) completed the same protocol.

### Data Analysis

Hand, bathtub rim, and bathtub seat pan positions were extracted using Cortex (Motion Analysis Inc). For EXIT, a single hand position was extracted, as participants did not remove and replace the hand during the transfer. For STS, a hand position was extracted for the STS direction, and a second hand position was extracted for the stand-to-sit direction. Positions were extracted in the vertical (VRT, I45, and I60) and horizontal (HH, LH, I45, and I60) directions, and along the hypotenuse of the grab bar (I45 and I60). All vertical positions were reported as distance above the bathtub rim. For EXIT, horizontal positions were reported as the distance from the center of the bathtub rim (negative for outside the bathtub, positive for inside the bathtub). For STS, horizontal and hypotenuse distances (for angled grab bars) were reported from the front edge of the bath seat (positive for in front of the bath seat, negative for behind the front edge of the bath seat).

Extracted data were analyzed using MATLAB (MathWorks). Hand position data were summarized for each grab bar and each task using heat maps. Separate ANOVAs were used to determine whether grasp location was affected by age (OA-HIP vs YA-HIP) or perturbation (YA-RTG perturbation vs no perturbation). In situations where there was only one grab bar for comparison (eg, VRT for vertical hand position during bathtub exit), a 1-way ANOVA was used. Where more than one grab bar was available (eg, VRT, I45, I60 for vertical position during STS), the grab bar was treated as a repeated measure, and age-grab bar or grasp-grab bar interactions were analyzed prior to main effects. Next, we evaluated whether grasp height (raw) was related to body height via correlation. For all statistical comparisons, a significance level (*α*) of .05 was used. Finally, position data were normalized to participant body height in order to make comparisons to the range of heights observed across the Canadian population.

## Results

Participant characteristics are summarized in Table 1.

**Table 1.** Participant characteristics.

Group	Protocol				
	EXIT			STS^a^	
	YA-HIP^b^	YA-RTG^c^	OA^d^	YA^e^	OA
Sex (n)					
Males	5	3	5	7	7
Females	5	7	6	10	10
Age (years), mean (SD)	24.6 (4.2)	23.7 (2.2)	67.7 (2.8)	24.5 (3.5)	71.6 (7.0)
Height (cm), mean (SD)	173.2 (11.8)	171.5 (9.0)	164.4 (8.2)	169.5 (9.8)	161.6 (9.0)
Weight (kg), mean (SD)	73.4 (15.5)	73.5 (14.2)	67.2 (12.1)	68.2 (16.3)	69.0 (14.5)

a STS: sit-to-stand.

b YA-HIP: younger adult, hand-in-place group.

c YA-RTG: younger adult, reach-to-grasp group.

d OA: older adult.

e YA: younger adult.

**Figure 1.**
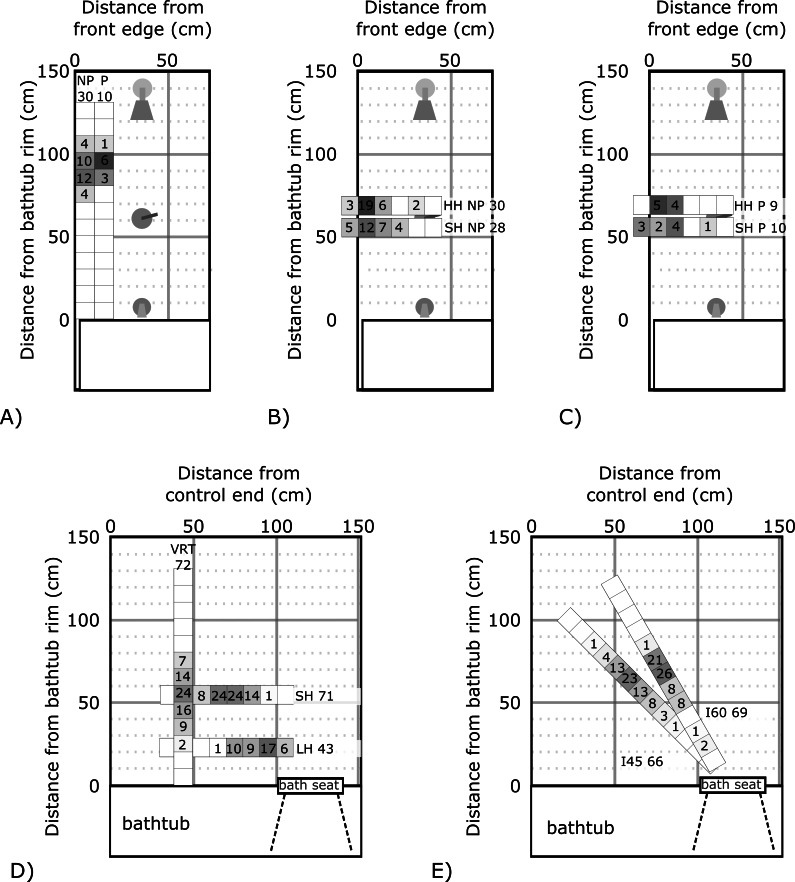
Heat map of grasp locations during EXIT and sit-to-stand. While only one perturbation and grab bar condition was tested during a single trial, multiple conditions are presented in each panel for comparison. Perturbation condition (Perturbation [P]; No perturbation [NP]) and grab bar label (**VRT, HH, SH, LH, I45, and I60**) are indicated to distinguish between conditions, along with the number of samples for each condition. Shading and number overlay indicate the number of participants who grasped in each location. (A) Vertical grab bar grasp locations during perturbation and non-perturbation exits. (B) Horizontal grab bar grasp locations during non-perturbation exits for HH and SH. (C) Horizontal grab bar grasp locations during perturbation exits for HH and SH. (D) Vertical and horizontal grab bar grasp locations during STS. (E) Incline grab bar grasp locations during sit-to-stand. HH: high horizontal; LH: low horizontal; SH: standard horizontal; VRT: vertical.

Across tasks and grab bars, vertical grasp locations ranged between 75.3 and 105.3 cm above the bathtub rim for EXIT, and 2.2 to 87.4 cm above the bathtub rim for STS. For STS, maximum grasp height always occurred during STS, and minimum grasp height always occurred during stand-to-sit. Horizontal grasp location ranged from 35.4 cm inside the bathtub rim to 7.6 cm outside the bathtub rim, for EXIT, and 6.1 cm behind the front edge of the bath seat to 43.5 cm in front of the front edge of the bath seat for STS. Along the hypotenuse of the angled grab bars, participants grasped as close to the front edge of the bath seat as 5.4 cm, and as far as 92.5 cm during STS. Grasp locations are depicted in Figure 1. Normalized grasp location data are summarized in Table 2.

**Table 2.** Grasp location descriptive statistics, normalized to participant body height.

Task and direction of measure	Grab bar	Grasp height		
		Maximum	Minimum	Mean (SD)
EXIT, perturbation^a^				
Vertical^b^	VRT^c^	76.3	67.1	73.0 (3.5)
Horizontal	SH^d^	18.5	−4.1^e^	5.3 (6.5)
Horizontal	HH^f^	6.7	0.2	4.0 (2.4)
EXIT, no perturbation				
Vertical^b^	VRT	83.0	70.2	76.4 (3.0)
Horizontal	SH	15.0	−3.2^e^	4.1 (4.5)
Horizontal	HH	22.4	−1.4^e^	5.3 (5.2)
STS^g^				
Vertical^b^	VRT	80.9	46.3	63.3 (7.8)
Horizontal	LH	20.4	−3.3^h^	6.1 (5.5)
Horizontal	SH	29.6	3.3	17.2 (5.8)
Vertical^b^	I60^i^	73.7	39.5	61.2 (7.7)
Horizontal	I60	18.7	−1.5^h^	10.7 (4.3)
Hypotenuse	I60	52.6	13.6	37.9 (7.8)
Vertical^b^	I45^j^	70.5	24.3	55.1 (9.0)
Horizontal	I45	20.0	0.8	11.9 (4.8)
Hypotenuse	I45	45.8	3.4	32.8 (8.7)

a Only young adult participants.

b Normalized grasp location includes 41cm bathtub rim height (ie, normalized grasp location represents height from the floor).

c VRT: vertical.

d SH: standard horizontal.

e Signifies a grasp location outside the midline of the bathtub rim.

f HH: high horizontal.

g Considers both sit-to-stand and stand-to-sit.

h Signifies a grasp location behind the front edge of the bath seat.

i I60: incline 60°.

j I45: incline 45°.

Grasp location did not differ between SH and HH grab bars during EXIT (Table 3). For STS, grasp height affected grasp location, such that participants grasped highest on the vertical grab bar, and on average, 5.9% lower on I60 than VRT, and 22.1% lower on I45 than VRT. The same trend held for stand-to-sit, with participants grasping at similar heights for VRT and I60, and grasping, on average, 21.3% lower for I45 than VRT. Participants grasped furthest away from the bath seat using SH, followed by I60 (33.6% closer than SH), I45 (28.3% closer than SH), and LH (63.1% closer than HH). No differences were observed between grasp locations along the hypotenuse of I60 and I45.

**Table 3.** Comparisons of grasp location between grab bars (ANOVA).

Task, direction of measure, and main effect factor	Grab bars	Interaction effect		Grab bar effect			Main effect	
		*F* test (*df*)	*P* value	*F* test (*df*)	*t* test	*P* value	*F* test (*df*)	*P* value
Exit								
Vertical								
Age	VRT^a^	—^b^	—	—	—	—	3.7 (1, 18)	.07
Horizontal								
Age	HH^c^ and SH^d^	−2.5 (1, 17)	.13	−1.6 (1, 17)	—	.23	−2.3 (1,17)	.15
Vertical								
Perturbation	VRT	—	—	—	—	—	11.6 (1,18)	<.01^e^
Horizontal								
Perturbation	HH and SH	−0.1 (1, 17)	.82	−0.14 (1, 17)	—	.72	−4.5 (1, 17)	.05^e^
STS								
Vertical max								
Age	VRT, I60^f^, I45^g^	1.3 (2, 60)	.28^h^	6.03 (2, 60)	—	<.01^e^^h^	0.0 (1, 30)	.968
Pairwise^i^	VRT and I60	—	—	—	3.9 (1)	<.01^e^	—	—
Pairwise	VRT and I45	—	—	—	6.8 (1)	<.01^e^	—	—
Pairwise	I60 and I45	—	—	—	5.2 (1)	<.01^e^	—	—
Vertical min								
Age	VRT, I60, and I45	2.1 (2, 60)	.13^h^	5.4 (2, 60)	—	<.01^e^^h^	0.4 (1, 30)	.51
Pairwise	VRT and I60	—	—	—	1.9 (1)	.06	—	—
Pairwise	VRT and I45	—	—	—	4.3 (1)	<.01^e^	—	—
Pairwise	I60 and I45	—	—	—	3.0 (1)	<.01^e^	—	—
Horizontal max								
Age	SH, LH^j^, I60, and I45	1.9 (3, 84)	.14	4.7 (3, 84)	—	.04^e^	0.0 (1, 28)	.95
pairwise	LH and SH	—	—	—	−12.8 (1)	<.01^e^	—	—
Pairwise	LH and I60	—	—	—	−4.8 (1)	<.01^e^	—	—
Pairwise	LH and I45	—	—	—	−6.7 (1)	<.01^e^	—	—
Pairwise	SH and I60	—	—	—	8.6 (1)	<.01^e^	—	—
Pairwise	SH and I45	—	—	—	5.8 (1)	<.01^e^	—	—
* *Pairwise	I60 and I45	—	—	—	2.3 (1)	<.01^e^	—	—
Horizontal min								
Age	SH, LH, I60, and I45	2.0 (3, 84)	.12	3.2 (3, 84)	—	.03	0.1 (1, 28)	.81
Pairwise	LH and SH	—	—	—	−10.6 (1)	<.01^e^	—	—
Pairwise	LH and I60	—	—	—	−3.5 (1)	.01^e^	—	—
Pairwise	LH and I45	—	—	—	−5.8 (1)	<.01^e^	—	—
Pairwise	SH and I60	—	—	—	5.5 (1)	<.01^e^	—	—
Pairwise	SH and I45	—	—	—	4.9 (1)	<.01^e^	—	—
Pairwise	I60 and I45	—	—	—	2.3 (1)	.03	—	—
Hypotenuse max								
Age	I60 and I45	0.6 (1, 30)	.45	0.2 (1, 30)	—	.62	0.1 (1, 30)	.75
Hypotenuse min								
Age	I60 and I45	0.0 (1, 30)	.99	0.6 (1, 30)	—	.45	0.0 (1, 30)	.96

a VRT: vertical.

b Not applicable.

c HH: high horizontal.

d SH: standard horizontal.

e Significant difference at *P*<.05.

f I60: incline 60°.

g I45: incline 45°.

h Outcome determined with Greenhouse-Geisser correction in consideration of violation of sphericity.

i Only significant pairwise comparisons reported.

j LH: low horizontal.

We did not observe any significant main effects of age, or age-grab bar interactions on grasp location.

A significant main effect of perturbation was observed, such that YA-RTG participants grasped 8.7 (6.6) cm, or, on average, 9.4% lower on the vertical grab bar during perturbation trials than nonperturbation trials (*F*_1, 18_=11.6, *P*<.01). However, perturbation was not a significant main effect for horizontal grab bars HH or LH (all *P*>.05).

Body height was significantly positively correlated with grasp height on vertical grab bars during nonperturbation EXIT (*r*=0.67, *P*<.01). Height was negatively correlated with maximum grasp distance during STS when using LH (*r*=−0.37, *P*=.03). No other significant correlations with height were observed. Grasp location normalized to body height is presented in Table 2.

## Discussion

### Principal Findings

In this study, we aimed to evaluate the grasp location on grab bars during 2 bathing tasks. We found that grasp location ranged vertically from approximately 70 cm above the bathtub rim, to just over a meter above the bathtub rim during bathtub exit. However, vertical grasp location was much more variable during the STS task, with grasp height ranging from just above the bathtub rim to nearly 90 cm above the bathtub rim. Horizontal grasp ranges were narrow for both tasks, with participants grasping within a range of 35 cm inside to just outside the bathtub for exit, and within 45 cm of the bath seat. Grasp location was affected by body height, grab bar location, and perturbation, but not age.

Participants preferred to grasp within 70%‐90% of their total body height (approximately shoulder height) during the unperturbed EXIT task, and slightly lower when their balance was perturbed during bathtub exit. A higher vertical grasp location above the COM is critical for the maintenance of balance during a transfer task. Komisar et al [20,25] demonstrated greater control of trunk kinematics following perturbation for a 42” high handrail compared with shorter handrails, as well as reduced loading on the grab bar. In comparison to the participants in this study, a 42” high grab bar would land between 54.9% and 68.4% of body height. Similarly, Greene et al [26] found better control of trunk flexion during unperturbed bathtub exit for a vertical grab bar than low grab bars mounted to the rim of the bathtub. Sveistrup et al [11] found that older adults preferred grab bar configurations with tall vertical grab bars for bathtub exit over grab bar configurations with a horizontal grab bar or no grab bar for the exit, with approximately 1/3 of participants stating that their ideal grab bar configuration would contain a vertical grab bar for entry and exit transfers. A long, vertical grab bar accommodates users of differing heights as well as supports bathtub entry/exit transfers with and without balance perturbations. Some shorter grab bars may also not be long enough to accommodate a user if installed considering only the preferred (unperturbed) grasp location. Further, grab bars that are installed based on preference may be too high to grasp during loss of balance.

Grasp height during STS was affected by grab bar configuration, with participants grasping higher with more vertical grab bars, and lower as the grab bar angle decreased. Similarly, the horizontal grasp location was further away from the bath seat for higher and more steeply angled grab bars, and closer for lower and less steeply angled grab bars. This may signify a change in grasp strategy, with participants using the VRT and higher grab bars as an apparatus to hang from, and using the lower and less steeply angled grab bars as an apparatus to push off from. While a higher grasp height may provide a greater balance advantage, during STS, this grasp height requires flexion of the arm above the shoulder; extending the arm above shoulder height may present a greater risk of injury [27], and may not be possible for some users with impairments to the shoulder or upper back. In contrast, a grasp location selected for a “push off” strategy may require less muscular effort from the upper extremity and reduce the risk of injury or discomfort with more conservative shoulder flexion. Dekker et al [28] similarly reported that during a toileting task, older adults prefer a push-off strategy when using horizontal grab bars between knee and shoulder height, and pulling up or hanging strategies when using vertical supports. In recreationally active young adults, upper body strength in a push-up position is 1.5‐2.7 times greater than that in a pull-up position [29]. While we did not observe any slips or falls in this study and did not measure balance outcomes, it may be valuable to understand the effect of pulling up compared with pushing off on balance metrics when using a grab bar for a STS task, as well as the impact of individual strength on selected grasp location and pull up or push off strategy. Additionally, the LH grab bar configuration we tested, specified in existing codes and standards [7,30] was below the preferred vertical grasp location for all but 2 participants, suggesting that the LH grab bar is inappropriately low for most people to use STS transfers.

We observed no change in horizontal grasp location between HH and SH during EXIT, and minimal changes in horizontal grasp location between SH, LH, I45, and I60 for STS. This echoes previous findings by Batista et al [12], who reported that participants most frequently grasped between 20 cm outside the bathtub rim and 20cm inside the bathtub rim during entry and exit. Several factors may contribute to this finding. First, participants may select a grasp location to minimize forward COM excursion during the grasping phase, grasping closer to their COM, and reducing the time between balance disturbance and hand position [31]. Second, closer grasp locations may require lower muscular effort [32]. Third, during a balance loss scenario, reaction time may be shorter for a closer grasp location, allowing more time for generating stabilizing forces. Finally, a closer grasp location may reflect the volume of visual field information stored for use during balance recovery reactions [19,33].

Considering these factors, our findings can be used to drive efforts to develop a universal grab bar configuration to support the bathing habits of most adults. Several existing codes and standards recommend a vertical grab bar for bathtub exit [7,8,30]. In our study, vertical grasp location on a VRT grab bar during bathtub entry and exit was moderately correlated with height, with participants grasping within 65%‐90% of their total body height, across EXIT conditions. Extrapolated across a range of heights from a 5th percentile female (142.45 cm) to a 95th percentile male (185.68 cm) [34], a minimum graspable area, including 10% tolerance, between 84 and 184 cm above the floor could be suggested for a grab bar placed at the entrance to a bathtub to support entry and exit. With the 41 cm rim height of the bathtub in this study, these dimensions translate to a graspable area between 43 and 143 cm above the bathtub rim. Positioning this vertical grab bar within 5 cm of the horizontal center of the bathtub rim will support the expected horizontal position of most users. For a grab bar along the back wall of the bathtub, participants of this study grasped vertically between 20% and 85% of their body height, which maps to a vertical graspable range of 25 to 156 cm above the floor (0 to 115 cm above the bathtub rim). Accommodating users of different heights may best be accomplished using a diagonal grab bar to span the vertical grasp range while allowing the user to select a preferred horizontal location. Higher grasp locations may be preferred to support other bathing tasks, such as washing the hair [35], while lower grasp locations further away from the control end of the bathtub may be appropriate for a bather who uses a bath seat or prefers to bathe seated at the bottom of the bathtub. We observed that participants prefer to grasp within a narrow horizontal range. Therefore, it may be valuable to consider the preferred grasp height of the user when considering the horizontal placement of ancillary items in the bathtub, such as a bath seat, soap dish, or towel rack, to allow continuous support along the varying heights of a diagonal grab bar.

There are cases where the suggested grab bar locations may not cover all use cases. In addition to evaluating grasp location during bathing tasks such as washing the feet or leaning over to test the water temperature, further work involving children may clarify whether the lower limits suggested are appropriate. Additionally, we only included participants who self-identified as healthy and able to bathe independently in this study; users with specific needs not covered by the suggested grab bar configurations, such as those with mobility challenges or who use mobility devices, may benefit from a more targeted or complex configuration of grab bars. For example, a horizontal grab bar may be preferred over a vertical grab bar by older adults [3,11] during bathtub entry and exit, STS from the bathtub floor, and balance recovery. In a study by Morales et al [36], older adults and users of mobility devices reported that using a vertical grab bar for bathing transfers felt less safe than a horizontal grab bar, while some users or participants refused to attempt to use the VRT grab bar to perform a bathing transfer, or reported that it felt uncomfortable or too physically challenging. Older adults and people who use mobility devices may place greater loads on a grab bar during a bathing transfer. Higher vertical loads may cause slipping on a vertical grab bar if they exceed the hand-grab bar friction generated through grip strength. Additionally, horizontal grab bars may allow the “push off” strategy, described previously, which may be more comfortable or require less physical effort than the “pull up” strategy required when using a higher grasp height on a vertical grab bar. Finally, upper extremity mobility, such as limited wrist or shoulder range of motion, may contribute to perceived comfort, effectiveness, or safety when using some grab bar configurations. Further work evaluating the use of grab bars during different bathing tasks with users of different mobility levels or assistive device requirements will inform optimal grab bar placement for other bathing subtasks and mobility levels.

### Limitations

This study had several limitations. First, to reduce the data collection burden, participants differed between EXIT and STS tasks. However, direct comparisons were not made between the 2 tasks. To maintain a safe data collection environment, we did not include a reactive grasping perturbation condition for older adults. While we did not observe an age effect for any outcome, age-related decreases in joint power [37,38] may amplify the decrease in grasp height we observed between nonperturbation and perturbation trials. We did not evaluate the shoulder range of motion, which has been demonstrated to decrease with age, and which may have influenced grasp height [24]. Third, we did not evaluate whole-body kinematics or kinetics in this study. Further exploration of this data may improve understanding of how preferred grasp location affects balance control and joint moments during bathing tasks. Fourth, we did not include children in our study population. While nearly 100% of children older than 12 years, and 25% of children aged 6‐11 years fall within the height range selected for our suggested universal configuration [34], further research could clarify whether the suggested configuration would also support children. It is possible that habitual higher grasps, used to reach grasping points on playground equipment, the hand of a caregiver, or to access handrails or surfaces, may affect overall grasping strategy and height in children. Fifth, it is unclear whether adaptation in reach-to-grasp strategies would impact grasp location. In a home bathroom, grasp location during habitual use and balance loss may converge. However, in an unfamiliar bathroom, such as in a hotel, grab bar positioning should consider both the proactive and reactive grasping locations observed in this study.

### Conclusions

In this study, we aimed to evaluate the grasp location of healthy adults during a bathtub exit task and a STS task. Preferred and reactive grasp locations were summarized to make suggestions for a universal grab bar configuration to support users of different heights. Based on experimental results, a 1-meter vertical grab bar in line with the bathtub rim will effectively support proactive grab bar use during bathing transfers, as well as reactive use during balance loss, for most Canadians. For STS from a bath seat, a diagonal grab bar will accommodate the change in grasp height between the STS and stand-to-sit phases of the task.

## Abbreviations

COM: center-of-mass
HH: high horizontal
I45: incline 45°
I60: incline 60°
LH: low horizontal
OA-HIP: older adult, hand-in- place group
SH: standard horizontal
SLS: sodium lauryl sulfate
STS: sit-to-stand
VRT: vertical
YA-HIP: younger adult, hand-in-place group
YA-RTG: younger adult, reach-to-grasp group
